# Characterization of the dynamics and the conformational entropy in the binding between TAZ1 and CTAD-HIF-1α

**DOI:** 10.1038/s41598-019-53067-8

**Published:** 2019-11-12

**Authors:** Ida Nyqvist, Jakob Dogan

**Affiliations:** Department of Biochemistry and Biophysics, Stockholm University, SE-10691 Stockholm, Sweden

**Keywords:** Chemical biology, Intrinsically disordered proteins

## Abstract

The interaction between the C-terminal transactivation domain of HIF-1α (CTAD-HIF-1α) and the transcriptional adapter zinc binding 1 (TAZ1) domain of CREB binding protein participate in the initiation of gene transcription during hypoxia. Unbound CTAD-HIF-1α is disordered but undergoes a disorder-to-order transition upon binding to TAZ1. We have here performed NMR side chain and backbone relaxation studies on TAZ1 and side chain relaxation measurements on CTAD-HIF-1α in order to investigate the role of picosecond to nanosecond dynamics. We find that the internal motions are significantly affected upon binding, both on the side chain and the backbone level. The dynamic response corresponds to a conformational entropy change that contributes substantially to the binding thermodynamics for both binding partners. Furthermore, the conformational entropy change for the well-folded TAZ1 varies upon binding to different IDP targets. We further identify a cluster consisting of side chains in bound TAZ1 and CTAD-HIF-1α that experience extensive dynamics and are part of the binding region that involves the N-terminal end of the LPQL motif in CTAD-HIF-1α; a feature that might have an important role in the termination of the hypoxic response.

## Introduction

Molecular recognition by proteins is an essential part of many cellular processes. While we have, during the last decades, greatly advanced our understanding on the molecular details of protein-ligand interactions, in particular the structural basis for such interactions, the significant role conformational entropy can have in molecular recognition is more recent^1–4^ and not as well understood. Nuclear magnetic resonance (NMR) spectroscopy has emerged as the most powerful experimental method for characterizing the internal motions of proteins at atomic resolution^5^. Indeed, recent studies have highlighted the crucial role dynamics at various time scales can have in many biological processes^1,6–13^.

**Figure 1:**
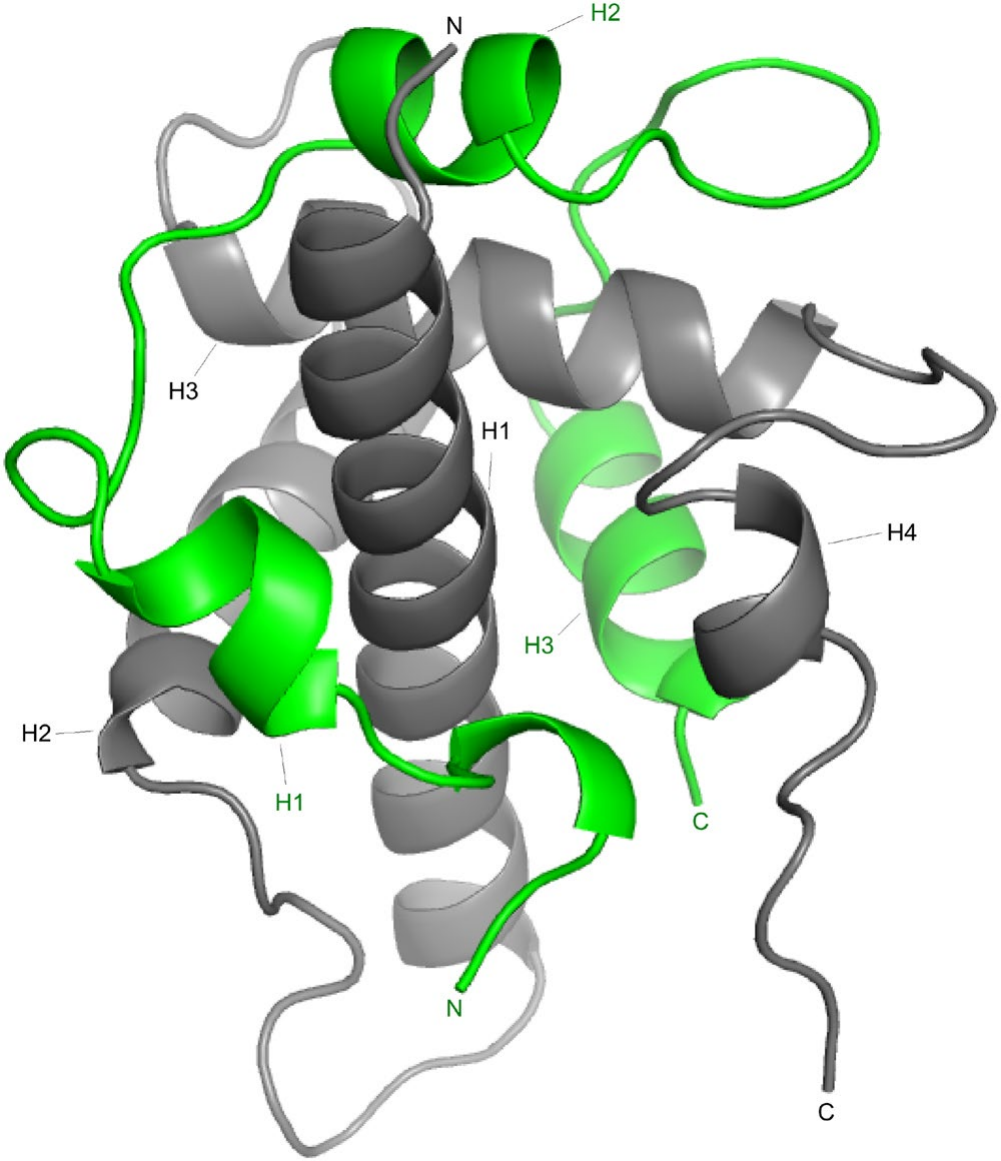
The 3D structure of the TAZ1/CTAD-HIF-1α complex^16^ (pdb code 1L8C). TAZ1 is shown in gray whereas CTAD-HIF-1α is shown in green. The helices in both TAZ1 and CTAD-HIF-1α are labeled.

We have, in this study, investigated the role of picosecond to nanosecond (ps-ns) dynamics in the interaction between the folded transcriptional adapter zinc binding 1 (TAZ1) domain of CREB binding protein (CBP)^14,15^ and the C-terminal transactivation domain of hypoxia inducible factor subunit 1α (CTAD-HIF-1α)^16,17^ by NMR. HIF-1α recruits CBP through the binding between TAZ1 and CTAD-HIF-1α, an interaction that plays a key role in the initiation of gene transcription that is crucial during hypoxia. At normoxic conditions this transcriptional regulation is tightly controlled through the hydroxylation of HIF-1α at certain positions^18^. During hypoxia however, hydroxylation no longer takes place, which protects HIF-1α from degradation, and CTAD-HIF-1α can now form tight interactions with TAZ1^16^, ultimately resulting in the activation of genes necessary for cell survival. It has also been shown that the nuclear protein, CITED2, acts as a negative feedback regulator of HIF-1α^19^. The solution three-dimensional NMR structures for free TAZ1^15^, and bound to CTAD-HIF-1α^16^ has been previously determined. TAZ1 is a four-helix bundle both in its free and bound state, whereas CTAD-HIF-1α wraps around TAZ1, forming three helices (Fig. 1). CTAD-HIF-1α is a so-called intrinsically disordered protein (IDP)^20^, which undergoes a coupled binding-and-folding reaction upon binding to TAZ1^16^. IDPs have during the last decade or so attracted attention due to their abundance in the eukaryotic proteome^20^, and their involvement in various cellular functions^14,20–22^. They have also been shown to be involved in many different types of diseases^23–25^. However, our understanding about the role of picosecond to nanosecond dynamics in the binding between a folded protein and the IDP remains rather limited, such as the extent of residual bound state dynamics, the motional response upon binding and its role in protein function. To what extent does the side chain and backbone dynamics of the folded protein change when interacting with different IDPs, and what does this mean in terms of energetic contribution to the overall binding thermodynamics? What is the dynamic character of the bound IDP? Thus, there is a need to perform studies that addresses the internal motions for both binding partners. Furthermore, many of the previously published NMR ps-ns dynamics studies on protein binding have mostly focused on backbone motions, but it has been demonstrated that it is crucial to also investigate side chain dynamics, which can correspond to substantial conformational entropy, are heterogeneous, are involved in allosteric phenomena, and can have a significant response upon binding^1,4,6,11,26,27^. However, studies in which the side chain fast dynamics have been investigated remain a minority among the experimental studies on protein dynamics by NMR.

We have here characterized the backbone amide and side chain methyl group ps-ns dynamics by ^15^N and ^2^H NMR relaxation, respectively. The side chain and the backbone dynamic response for the well-folded TAZ1 upon binding to CTAD-HIF-1α corresponds to a conformational entropy change that contributes significantly to the binding thermodynamics. Furthermore, side chain methyl group dynamics for bound CTAD-HIF-1α reveals that a certain region still experience extensive motion while the dynamics of other regions is more restricted. Comparison of the dynamics in TAZ1/CTAD-HIF-1α with the previously characterized binding between TAZ1 and the transactivation domains of STAT2 (TAD-STAT2)^28^ and RelA (RelA-TA2)^29^ demonstrates that the side chain and backbone motional response of TAZ1 varies depending on the target. Finally, the analysis of side chain dynamics reveals the presence of a cluster consisting of side chains from both TAZ1 and CTAD-HIF-1α that are highly dynamic and are part of a certain binding region that is important in the competition for TAZ1 binding between CTAD-HIF-1α and the CITED2 protein.

## Results and Discussion

The aim of this study was to elucidate the role of picosecond to nanosecond dynamics for both binding partners in the interaction between TAZ1 and CTAD-HIF-1α. For this purpose, backbone ^15^N-relaxation and side chain methyl ^2^H-relaxation experiments were carried out for bound TAZ1 and side chain methyl ^2^H-relaxation for bound CTAD-HIF-1α. CTAD-HIF-1α has previously been shown to be completely disordered in its free state but undergoes a disorder-to-order transition upon binding to TAZ1^16^. TAZ1 is a folded protein domain^15^ and the equilibrium dissociation binding constant for this interaction is in the low nanomolar region as reported earlier^16,30^.

### Backbone dynamics

**Figure 2:**
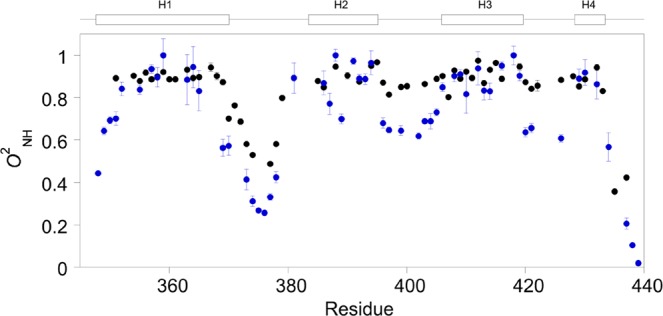
Backbone *O*^2^_NH_ parameters for bound (black) and free TAZ1 (blue). The *O*^2^_NH_ values for free TAZ1 are reprinted with permission from ref.^28^. Copyright 2018 American Chemical Society. The helical regions are shown above the graph.

Model-free analysis^31^ of backbone ^15^N-relaxation data provides the squared generalized order parameter for the amide group, *O*^2^_NH_, which reports on the amplitude of motions that are expressed on the ps-ns time scale. *O*^2^_NH_ adopts values between zero, reflecting completely unrestricted motions, and one, which reports on complete rigidity. The average *O*^2^_NH_ for the helices in TAZ1 is 0.89 ± 0.06, which reflects restricted motions at these regions (Fig. 2, Supplementary Table S1). One exception is the C-terminal end of helix one, which experience more mobility, but is less pronounced compared to the free state of TAZ1 (Supplementary Table S2)^28^. The loop between helix one and two experiences extensive ps-ns motions, whereas the other two loops in TAZ1 become rigidified in the bound state (Fig. 2). This behavior is different compared to when TAZ1 is bound to a different target, TAD-STAT2, where TAZ1 overall retains its backbone amide group ps-ns dynamics^28^. The CTAD-HIF-1α-bound TAZ1 is also more rigid when compared to TAZ1 bound to RelA-TA2^29^, which also experiences a rigidification but not to the same extent as in TAZ1/CTAD-HIF-1α. Thus, this demonstrates that TAZ1, although a well-folded protein domain, can have a backbone dynamic response that varies depending on the binding partner.

### Side chain dynamics of bound TAZ1 and bound CTAD-HIF-1α

**Figure 3:**
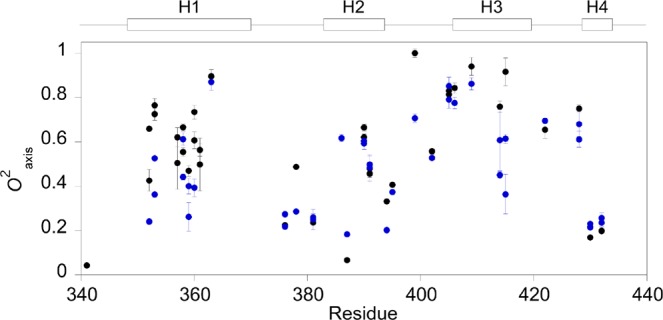
The dynamic character of methyl-bearing residues in TAZ1. Side chain methyl axis order parameters, *O*^2^_axis_, are here shown for bound (black) and free (blue) TAZ1^28^. The *O*^2^_axis_ values for free TAZ1 are reprinted with permission from ref.^28^. Copyright 2018 American Chemical Society. The helical regions are shown above the graph.

**Figure 4:**
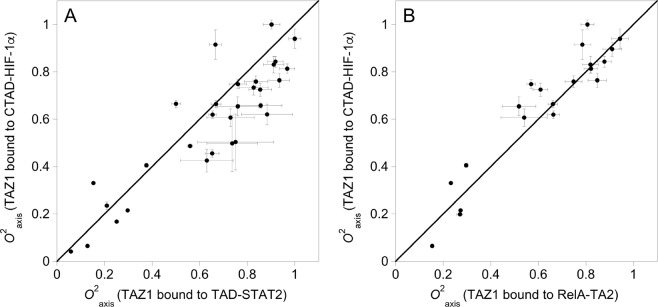
Site-to-site comparison of *O*^2^_axis_ parameters when TAZ1 is bound to (**A**) TAD-STAT2^28^ (horizontal axis) and CTAD-HIF-1α (vertical axis), and (**B**) RelA-TA2^29^ (horizontal axis) and CTAD-HIF-1α (vertical axis). Data points that are positioned to the right side of the diagonal line corresponds to higher mobility for that methyl group in the TAZ1/CTAD-HIF-1α complex than in the TAZ1/TAD-STAT2 or TAZ1/RelA-TA2 complex. The *O*^2^_axis_ values for TAD-STAT2-bound TAZ1 are reprinted with permission from ref.^28^. Copyright 2018 American Chemical Society. The *O*^2^_axis_ values for RelA-TA2-bound TAZ1 are reprinted with permission from ref.^29^. Copyright 2019 American Chemical Society.

Side chain picosecond to nanosecond methyl group dynamics was investigated by deuterium relaxation for bound TAZ1. We were able to determine the model-free squared generalized methyl axis order parameter, *O*^2^_axis_, and the effective internal correlation time,* τ*_e_, for 36 methyl groups (Fig. 3, Supplementary Table S3) of a total of 51. For the remaining methyls the peaks were either overlapped or had too weak intensities and therefore prohibited further analysis. As shown in Fig. 3, the dynamics for methyl–bearing residues is quite heterogeneous, in line with previous reports on protein side chain dynamics^32–36^. The binding interface of TAZ1/CTAD-HIF-1α contains several methyls, with *O*^2^_axis_ values ranging from 0.066 (Met-387ε) to 0.76 (Ile-353γ2). Overall, the methyl groups are more dynamic compared to those observed for TAZ1 bound to TAD-STAT2^28^ (Fig. 4A), and similar when compared to the RelA-TA2-bound TAZ1 (Fig. 4B)^29^.

**Figure 5:**
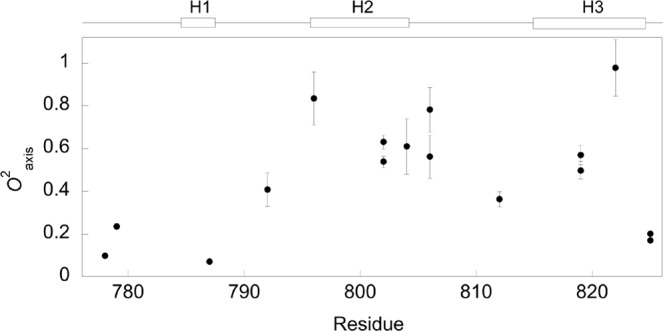
Side chain methyl axis order parameters for bound CTAD-HIF-1α. The valine and leucine methyls are not stereospecifically assigned. The helical regions are shown above the graph.

Side chain subnanosecond dynamics of bound CTAD-HIF-1α was also studied by ^2^H relaxation, from which we determined the *O*^2^_axis_ parameter for 16 methyl groups (Fig. 5, Supplementary Table S4). Figure 5 shows that the methyl groups in the N-terminal region of CTAD-HIF-1α are highly dynamic, whereas higher *O*^2^_axis_ values are later generally adopted, up to residue Leu-822 with TAZ1. This resonates well with the backbone dynamics studies previously reported for bound CTAD-HIF-1α to TAZ1^37,38^, which also showed that, even though the N-terminal part of CTAD-HIF-1α makes direct interactions with TAZ1, it is highly dynamic on the ps-ns time scale^37,38^, a region which has been characterized as fuzzy^38–42^. Leu-812, which is part of the dynamic loop between helices two and three also experience extensive motions on the side chain level with the *O*^2^_axis_ value for Leu-812δ2 equal to 0.36 ± 0.04.

**Figure 6:**
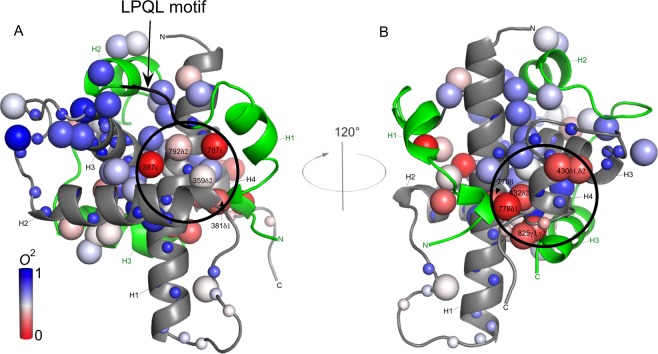
The dynamic character of the TAZ1/CTAD-HIF-1α complex. The backbone amide and side chain methyl groups for which the order parameter could be determined for bound TAZ1 and CTAD-HIF-1α are here represented as spheres which are color-coded according to the order parameter value from zero to one using a red-white-blue gradient. Large spheres represent the methyl groups whereas the smaller spheres represent the main-chain amide groups. The backbone of TAZ1 is colored gray, whereas CTAD-HIF-1α is colored green. (**A)** The backbone of the LPQL motif is colored black, and the dynamic cluster that is close to this motif is encircled. (**B)** The other cluster is here encircled, which involves the dynamic methyl groups that are part of the C-terminal end of TAZ1 and the N- and C-terminals of CTAD-HIF-1α.

It has been previously reported that CITED2 competes with HIF-1α for TAZ1 binding in a highly efficient manner. A key player in this process is the LP(Q/E)L motif that is present in CTAD-HIF-1α and also in the carboxyl-terminal transactivation domain of CITED2 (CTAD-CITED2), where it binds at the same region of TAZ1^37^. It is therefore of interest to inspect the dynamic nature of the LPQL motif in CTAD-HIF-1α. We were ™able to determine the *O*^2^_axis_ parameter for Leu-792δ2, which is rather low (*O*^2^_axis_ = 0.41) and suggests an increased mobility for this side chain relative to those that were determined in the more ordered region of CTAD-HIF-1α. This agrees well with the backbone dynamics for Leu-792 as reported by Berlow *et al*.^37,38^. In Fig. 6, the order parameters for bound TAZ1 and CTAD-HIF-1α are mapped onto the protein complex, which shows that there are two dynamic clusters (Fig. 6A,B). One of these clusters involves highly dynamic methyl groups that are part of the C-terminal end of TAZ1 and the N- and C-terminals of CTAD-HIF-1α (Fig. 6B). Interestingly, the other dynamic cluster (Fig. 6A) involves the side chain methyl groups Leu-359δ2, Leu-381δ1, and Met-387ε in bound TAZ1, and Met-787ε, and Leu-792δ2 in bound CTAD-HIF-1α. Leu-359δ2, Leu-381δ1, and Met-387ε are already dynamic in the free state but retain their mobility in the bound state to a large extent, with Met-387ε even having higher mobility in the bound state. It is thus tempting to speculate that the dynamic nature of this region, which includes the beginning of the LPQL binding site, is important for the facilitation of the displacement of CTAD-HIF-1α by CTAD-CITED2. Furthermore, it has been previously suggested^37^ that CTAD-CITED2 initially binds the CTAD-HIF-1α-bound TAZ1 in the region that interacts with the highly dynamic N-terminal part of CTAD-HIF-1α that precedes the LPQL motif, leading to the formation of a transient ternary complex, which would result in structural changes in TAZ1 that lowers the affinity to CTAD-HIF-1α, and ultimately leading to its complete displacement from TAZ1. Thus, the presence of the cluster of dynamic side chains at the N-terminal end of this binding region might make it easier to displace CTAD-HIF-1α, suggesting an important role for conformational entropy in negative feedback regulation. However, future studies needs to be performed in order to further investigate the relationship between the bound state dynamics and the displacement mechanism.

### Changes in internal motions upon binding

**Figure 7:**
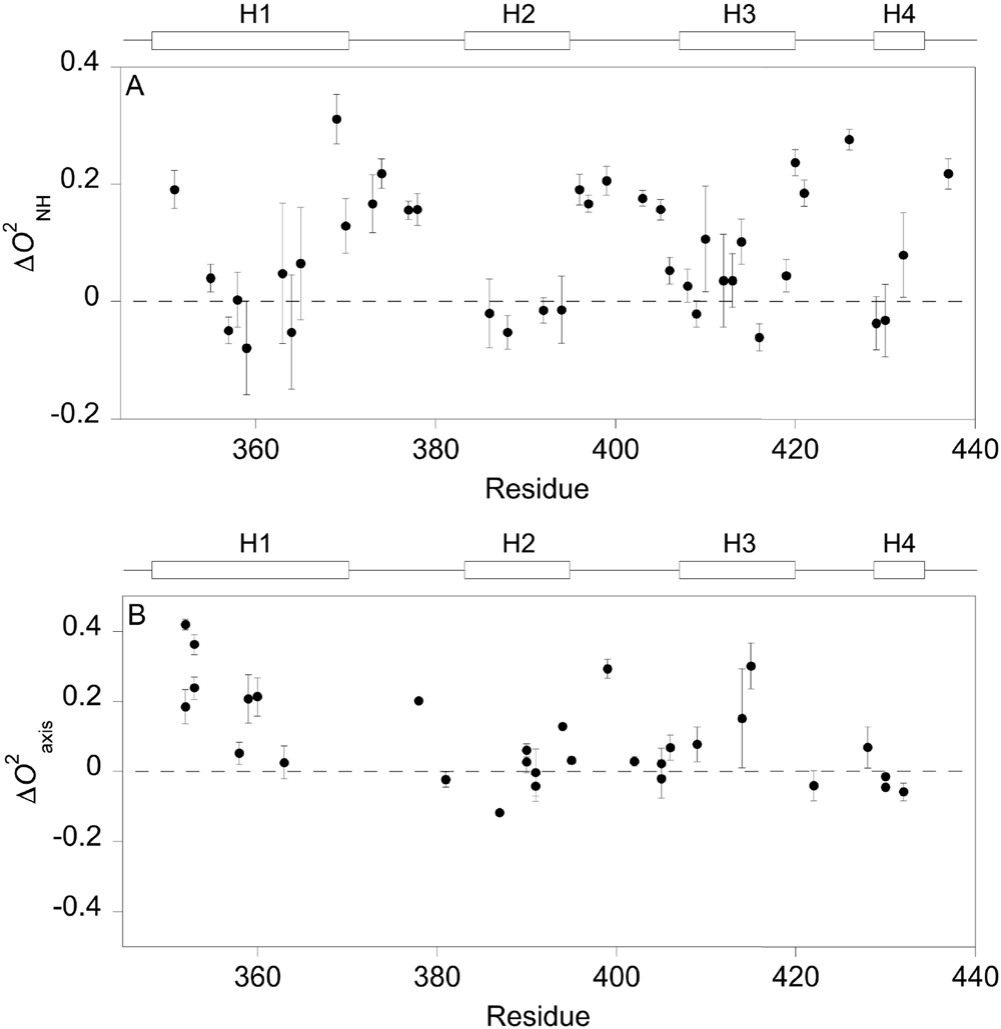
The dynamic response for TAZ1 upon binding to CTAD-HIF-1α. Differences between (**A)** the backbone *O*^2^_NH_ parameters, and (**B)** the side chain *O*^2^_axis_ parameters of bound and free TAZ1 vs residue number.

**Figure 8:**
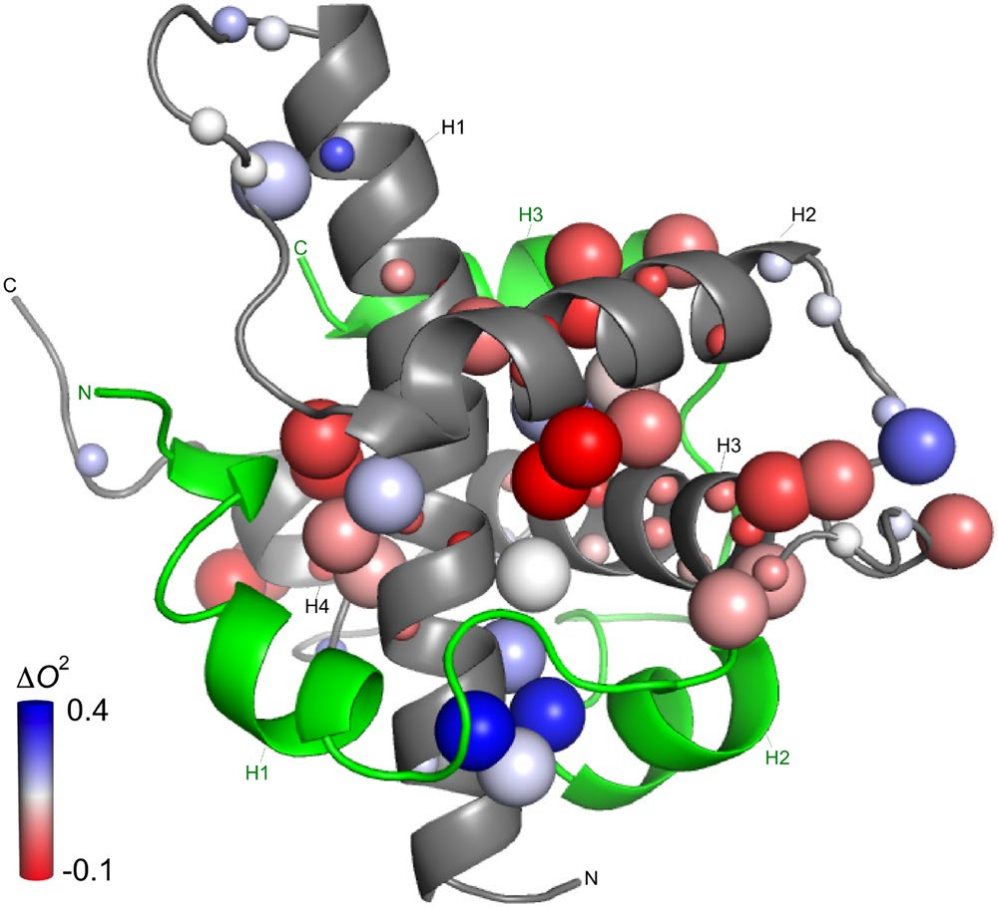
Changes in order parameters (both Δ*O*^2^_NH_ and Δ*O*^2^_axis_) for TAZ1 upon binding to CTAD-HIF-1α are color-coded using a red-white-blue gradient. Large spheres represent the methyl groups and the smaller spheres represent the backbone amide groups. The backbone of TAZ1 is shown in gray, and CTAD-HIF-1α in green.

The backbone dynamic response for TAZ1 upon binding CTAD-HIF-1α is shown in Fig. 7A. Overall, the main-chain of TAZ1 becomes rigidified on the picosecond to nanosecond time scale. The pairwise mean difference in *O*^2^_NH_ for TAZ1 is equal to 0.086 ± 0.017 (mean ± standard error of the mean), a result which is corroborated by a recent study by Berlow *et al*.^38^. This can be compared to a value of 0.005 ± 0.013 for the dynamic response for TAZ1 upon binding to TAD-STAT2^28^ and 0.040 ± 0.016 for the binding to RelA-TA2^29^, which clearly shows that the backbone internal motions vary between different IDP targets. The backbone of the three loops in TAZ1 are less dynamic in the bound state (Figs 7A and 8) with *O*^2^_NH_ changes for these loops being of similar size, although the loop between helix one and two still experience extensive dynamics. One likely reason for the rigidification of the loop between helix two and three in the TAZ1/CTAD-HIF-1α complex is that this loop makes numerous contacts with CTAD-HIF-1α, whereas in the TAZ1/TAD-STAT2 complex, this loop only forms very few contacts^43^ with the highly disordered^28^ C-terminal part of TAD-STAT2. A stiffening of the same loop was also observed to take place in the TAZ1/RelA-TA2 complex^29^, a region where TAZ1 makes several contacts with RelA-TA2. It seems that the differences in loop dynamics upon binding is one important reason for the observed motional variations among the TAZ1/target associations. The largest dynamic changes on the side chain level are seen for methyls Leu-352δ1,δ2, Ile-353δ1,γ2, Leu-359δ2, Leu-360δ1, Ala-378β, Met-387ε, Met-394ε, Ala-399β, Ile-414γ2, and Ile-415γ2 (Figs 7B and 8), all of which become more rigid upon binding to CTAD-HIF-1α (positive ∆*O*^2^_axis_ values in Fig. 7B corresponds to increased rigidity), with the exception for Met-387ε (∆*O*^2^_axis_ = −0.12 ± 0.01), which is more mobile in the bound state. Almost all of these methyls (residues 378 and 399 are distant to the interaction surface) make direct interactions with CTAD-HIF-1α. The region in TAZ1 encompassing residues 352–360 and 414–415, which includes most of the methyl-bearing residues that experience the largest dynamic changes in TAZ1 upon binding to CTAD-HIF-1α (Fig. 7B), were also found to be the regions in TAZ1 of which the motional response were the greatest in the interaction with TAD-STAT2 or RelA-TA2. Thus, while these residues form important enthalpic interactions with the binding partner, it appears that they also substantially affect the thermodynamics of association by means of a conformational entropy change. The pairwise mean difference in *O*^2^_axis_ is 0.094 ± 0.024 for TAZ1 upon binding to CTAD-HIF-1α. Thus, there is a reduction in ps-ns motions both on the backbone and side chain level for TAZ1. The overall change in methyl group dynamics in TAZ1 is smaller when compared to the binding between TAZ1 and TAD-STAT2^28^, which resulted in a 〈∆*O*^2^_axis_〉 of 0.155 ± 0.030, and similar to that of TAZ1/RelA-TA2^29^ with 〈∆*O*^2^_axis_〉 = 0.073 ± 0.022. This demonstrates that the backbone and the side chains in TAZ1 can have different dynamic responses depending on the target.

Previous studies have established relationships between changes in order parameters and the change in protein conformational entropy, Δ*S*_conf_^1,4,44–48^. We have here used these relationships^4,49^ and by using the average pairwise differences in the order parameters, we determined the conformational entropy change (−TΔ*S*_conf_) for TAZ1, and found it to be equal to 23.5 kcal/mol that is contributed from the backbone and 6.6 kcal/mol from the side chains, resulting in a total change of 30.1 kcal/mol for TAZ1. Thus, this demonstrates that the conformational entropy change makes a substantial contribution to the binding thermodynamics, and in this instance it is unfavorable for complex formation, which was also shown to be the case for the binding between TAZ1 and TAD-STAT2^28^. However, in TAZ1/TAD-STAT2 (Δ*G*_bind_ = −10.2 kcal/mol), most of the conformational entropy change in TAZ1 came from the side chains (11 kcal/mol contributed by the side chains and 0.9 kcal/mol by the main-chain), which is different for TAZ1/CTAD-HIF-1α. Here it is the backbone that makes the largest contribution to −T∆*S*_conf_(TAZ1). In the case of TAZ1/RelA-TA2^29^ (Δ*G*_bind_ = −10.1 kcal/mol), there were significant contributions to the conformational entropy change from both the backbone (5.1 kcal/mol) and the side chains (9.9 kcal/mol) in TAZ1. This clearly shows that the microscopic details of the motional response in TAZ1 and the conformational entropy change that it represents depend on the target. TAZ1 has been previously characterized as a rigid scaffold for binding^15^. However, this work shows together with previous recent studies^28,29,38^ that the ps-ns dynamics of TAZ1 have crucial ramifications for the interactions. The contribution from the conformational entropy change even exceeds in magnitude compared to the Gibbs free energy change (~−11.5 kcal/mol) for TAZ1/CTAD-HIF-1α, which shows that various different energetic contributions are at play. Since we have also determined *O*^2^_axis_ parameters for methyl groups in bound CTAD-HIF-1α (Supplementary Table S4), and while it would have been desirable to have more probes in the dynamic N-terminal region of CTAD-HIF-1α, we can make an estimation of how much the side chain conformational entropy change may contribute to the binding energetics, assuming that the *O*^2^_axis_ parameter in the free state of CTAD-HIF-1α is uniform and adopts a value of 0.05^2,4^ (since unbound CTAD-HIF-1α is completely disordered). This results in a side chain conformational entropy change of about 14 kcal/mol for CTAD-HIF-1α, which demonstrates the potential of the conformational entropy change both for the folded and the disordered binding partner of being an important player in the binding thermodynamics. Without consideration of such energetic contributions to the free energy of binding of either partner would result in a biologically meaningless binding affinity.

## Conclusions

We have here shown the importance of fast time scale dynamics for the binding between TAZ1 and CTAD-HIF-1α. Both backbone and side chain motions are affected to a significant extent upon binding. This dynamic response for both binding partners corresponds to a conformational entropy change that substantially affects the binding thermodynamics. Furthermore, comparison to two previously characterized TAZ1/IDP interactions^28,29^ shows that the side chain and backbone internal motions of TAZ1 vary significantly depending on the target. We also observe that there is a dynamic cluster in the complex, which might have an important role in the competition for TAZ1 binding between CTAD-HIF-1α and CTAD-CITED2.

## Materials and Methods

### Protein expression and purification

TAZ1 (residues 340–439; this numbering is according to the mouse CBP sequence, and this specific TAZ1 sequence is the same in the corresponding region in human CBP, and the mouse CBP numbering was also used in previous studies^28–30,50^) and CTAD-HIF-1α (residues 776–826) were expressed and purified as previously described^28,30,50^. ^15^N-labeled TAZ1 was expressed in M9 minimal medium supplemented with ^15^NH_4_Cl. ^15^N, ^13^C, and ^2^H-labeled proteins were produced in M9 minimal medium that contained 60% D_2_O and was supplemented with ^15^NH_4_Cl and ^13^C-glucose (99% U-^13^C_6_). TAZ1 was also expressed in minimal medium that contained 10% ^13^C-glucose and 90% unlabeled glucose in order to obtain a NMR sample for the stereospecific assignment of methyls in valines and leucines^51^.

### NMR samples

All NMR experiments were performed using samples that contained 15 mM HEPES (pH = 6.8), 50 mM NaCl, 3 mM TCEP, 0.01% NaN_3_, and 7% D_2_O. Backbone relaxation experiments were carried out on a sample that contained 1.3 mM ^15^N TAZ1/1.8 mM unlabeled CTAD-HIF-1α, and for side chain relaxation measurements, samples containing 1.3 mM ^2^H, ^13^C, ^15^N-TAZ1/1.8 mM unlabeled CTAD-HIF-1α, and 1.3 mM ^2^H, ^13^C, ^15^N-CTAD-HIF-1α/1.8 mM unlabeled TAZ1 were used. We also prepared a sample that contained 0.5 mM 10% ^13^C-TAZ1/0.7 mM unlabeled CTAD-HIF-1α in order to obtain the stereospecific assignments of the valine and leucine methyls in bound TAZ1^51^.

### NMR experiments

NMR experiments were performed using Bruker NMR spectrometers operating at Larmor ^1^H frequencies of 500, 600, and 700 MHz, at a temperature of 303 K. ^15^N backbone *R*_2_, *R*_1_, and {^1^H}−^15^N heteronuclear nuclear Overhauser effect (NOE) experiments for bound TAZ1 were carried out at 700 MHz (Supplementary Fig. S1). In the ^15^N *R*_2_, *R*_1_ experiments 8–12 time points with two to three of them being duplicates in order to assess the uncertainty in peak intensities were acquired. Time points in the *R*_2_ measurement ranged from 16.6 ms to 116.3 ms, while for *R*_1_ they were between 0.02 s and 1.40 s. For the side chain relaxation experiments (I_z_C_z_D_z_, I_z_C_z_D_y_, and I_z_C_z_)^52^ eleven to twelve time points, including three duplicates were acquired for both bound TAZ1 and bound CTAD-HIF-1α at both 700 and 500 MHz (Supplementary Tables S5 and S6). At 700 MHz and for bound TAZ1, these were in the range 1.6–64.6 ms (I_z_C_z_D_z_), 0.4–11.1 ms (I_z_C_z_D_y_), and 1.6–65.1 ms (I_z_C_z_), whereas for bound CTAD-HIF-1α the ranges were 1.6–55.1 ms (I_z_C_z_D_z_ and I_z_C_z_), and 0.4–15.0 ms (I_z_C_z_D_y_). At 500 MHz they ranged from 1.6 ms to 55.1 ms (I_z_C_z_D_z_ and I_z_C_z_), and 0.4 ms to 13 ms (I_z_C_z_D_y_) for complexed CTAD-HIF-1α, while for bound TAZ1 they were between 1.6 ms and 58.6 ms (I_z_C_z_D_z_ and I_z_C_z_), and 0.4–11.1 ms for I_z_C_z_D_y_. The stereospecific assignments for the valine and leucine methyls for bound TAZ1 were determined by acquiring a constant-time ^13^C-HSQC spectrum at 700 MHz on the 10% ^13^C labeled bound TAZ1.

### Relaxation analysis

NMR data were processed using NMRPipe^53^. Backbone ^15^N *R*_1_, *R*_2_, and {^1^H}−^15^N NOE data for bound TAZ1 were subjected to model-free analysis^31^ using the program suite *relax*^54,55^. An initial estimation of the global tumbling was determined from the *R*_2_/*R*_1_ ratio, using only residues with a heteronuclear NOE value greater than 0.65, and by also removing residues that experience conformational exchange^56,57^. Five different local models^58^ for each residue were optimized. Model selection as well as diffusion tensor selection was carried out using the Akaike information criterion^54,55^. An axially symmetric diffusion tensor was selected as the best fit, with a global correlation time equal to 10.7 ns and an anisotropy, D_∥_/D_⊥_, of 1.09. Errors in model-free parameters were obtained using 500 Monte Carlo simulations. Collection of relaxation data and subsequent implementation of Lipari–Szabo model-free type analysis as used for the TAZ1/CTAD-HIF-1α complex was not performed for free CTAD-HIF-1α since such analysis is problematic due to the underlying assumption in standard model-free analysis that global and internal motions are statistically independent, and a single global correlation time describing the free IDP, which is suspect. However, recent studies have developed extended methodologies for the examination of internal dynamics in IDPs^59^.

The ^2^H D_z_ and D_y_ rates for bound TAZ1 and CTAD-HIF-1α were determined through subtraction of the I_z_C_z_ rate from the I_z_C_z_D_z_ and I_z_C_z_D_y_ rates, respectively. These rates were subsequently used to obtain model-free squared generalized order parameters and effective internal correlation times^32,52^, in which the global correlation time determined from the ^15^N backbone relaxation data was used, and a ^2^H quadrupolar coupling constant of 167 kHz^60^ was set in the calculations. The squared generalized methyl axis order parameters, *O*^2^_axis_, were determined by taking the motion about the methyl symmetry axis into consideration. Uncertainties were determined by 500 Monte Carlo simulations. Total backbone NH, and side chain conformational entropy changes were calculated as previously described^4,49^.

## Supplementary information

Supplementary Information
