# Supplementary material for: Characterization of the dynamics and the conformational entropy in the binding between TAZ1 and CTAD-HIF-1α
Source: Sci Rep. 2019 Nov 12;9:16557. doi: 10.1038/s41598-019-53067-8 (PMC6851107; doi:10.1038/s41598-019-53067-8)
Supplement: Supplementary file 1 — Supplementary Information [file 41598_2019_53067_MOESM1_ESM.pdf]

Supplementary Information

Characterization of the dynamics and the conformational entropy in the binding between TAZ1 and CTAD-HIF-1α

Ida Nyqvist and Jakob Dogan*

Department of Biochemistry and Biophysics, Stockholm University, SE-10691 Stockholm, Sweden

*Corresponding author: email: jakob.dogan@dbb.su.se, telephone: +46-8-162470

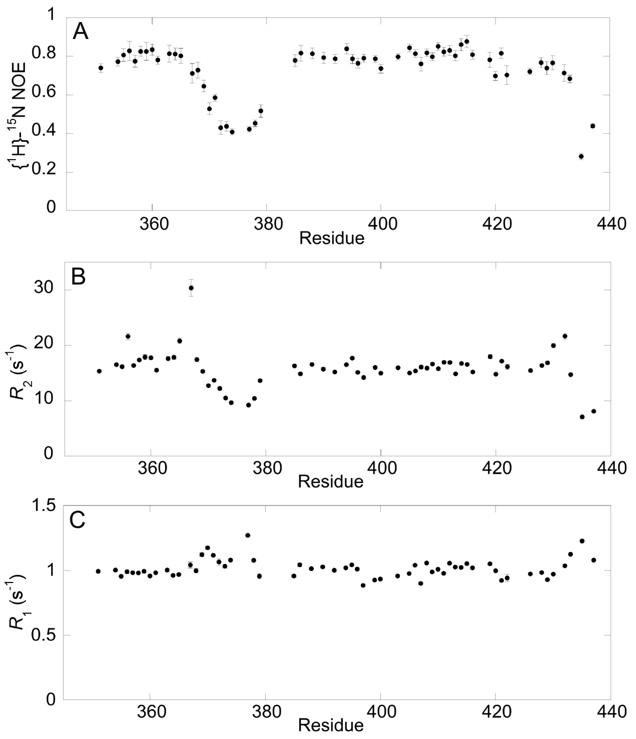

Figure S1. Backbone relaxation data for TAZ1 bound to CTAD-HIF-1α, at 700 MHz. A) $\{^1H\}-^{15}N$ NOE B) ^{15}N R_2, and C) ^{15}N R_1.

Table S1. Model-free parameters for TAZ1 bound to CTAD-HIF-1α.

Residue	O^2_{NH}	Error	τ_e (ns)	Error	R_{ex} (s^{-1})	Error	Model
351	0.89	0.01	0.04	0.01			2

354	0.90	0.01	0.03	0.01	1.0	0.2	4
355	0.88	0.01			1.1	0.3	3
356	0.92	0.01			5.7	0.7	3
357	0.89	0.01	0.03	0.01	1.2	0.3	4
358	0.90	0.01			2.0	0.2	3
359	0.92	0.01			2.0	0.5	3
360	0.89	0.01			2.5	0.3	3
361	0.89	0.01	0.02	0.01	0.4	0.2	4
363	0.93	0.01			1.4	0.5	3
364	0.89	0.01			2.4	0.3	3
365	0.90	0.01			5.3	0.5	3
367	0.94	0.02	0.12	0.12	13.9	1.6	4
368	0.90	0.02	0.05	0.02	1.8	0.5	4
369	0.87	0.01	0.67	0.10			5
370	0.70	0.01	0.86	0.06			5
371	0.76	0.01	0.81	0.04			5
372	0.69	0.01	0.63	0.06			5
373	0.58	0.01	0.77	0.04			5
374	0.53	0.01	0.83	0.02			5
377	0.49	0.00	0.98	0.02			5
378	0.58	0.01	0.82	0.03			5
379	0.80	0.01	0.07	0.01			2
385	0.88	0.01	0.02	0.01	1.0	0.3	4
386	0.85	0.02	1.87	1.35			5
388	0.95	0.01					1
390	0.91	0.02	0.78	0.74			5
392	0.88	0.01	0.76	0.38			5
394	0.95	0.01					1
395	0.97	0.01	0.09	0.07	0.7	0.3	4
396	0.87	0.01	0.69	0.25			5
397	0.82	0.01	0.01	0.00			2
399	0.85	0.01	0.02	0.00	1.2	0.2	4
400	0.86	0.01	0.03	0.01			2
403	0.87	0.01	0.01	0.00	1.2	0.1	4
405	0.89	0.01					1
406	0.90	0.01	1.02	0.82			5
407	0.80	0.01	0.02	0.01	2.4	0.3	4
408	0.93	0.01	0.94	1.02			5
409	0.89	0.01	0.02	0.01	1.5	0.2	4
410	0.92	0.00					1
411	0.89	0.01			1.8	0.2	3
412	0.98	0.01					1

413	0.87	0.01	1.09	0.58			5
414	0.93	0.01			0.9	0.3	3
415	0.97	0.01					1
416	0.89	0.01	0.89	0.82			5
419	0.95	0.01	0.06	0.03	1.8	0.4	4
420	0.87	0.01	0.05	0.01			2
421	0.84	0.01			2.9	0.3	3
422	0.86	0.03	0.04	0.01	1.1	0.6	4
426	0.88	0.00	0.05	0.00			2
428	0.90	0.01	0.03	0.01	0.7	0.2	4
429	0.85	0.01	0.03	0.01	1.8	0.4	4
430	0.89	0.01	0.03	0.01	4.6	0.4	4
432	0.94	0.02	0.12	0.05	5.1	0.6	4
433	0.83	0.01	0.92	0.08			5
435	0.36	0.00	0.90	0.02			5
437	0.42	0.00	1.00	0.01			5

Table S2. Model-free parameters for free TAZ1. Reprinted with permission from ref 1. Copyright 2018 American Chemical Society.

Residue	O^2_{NH}	Error	τ_e (ns)	Error	R_{ex} (s^{-1})	Error	Model
348	0.44	0.01	1.11	0.03			5
349	0.64	0.02	0.96	0.09			5
350	0.69	0.02	0.55	0.14			5
351	0.70	0.03	0.76	0.27			5
352	0.84	0.03	0.04	0.02	1.2	0.4	4
355	0.84	0.02	0.03	0.01			2
357	0.94	0.02	0.10	0.21			2
358	0.90	0.05	0.04	0.28	2.3	0.7	4
359	1.00	0.08			4.3	1.7	3
363	0.89	0.12	1.50	1.35	4.5	1.4	4
364	0.95	0.10	0.47	0.99	7.2	1.9	4
365	0.83	0.10	0.02	0.45	12.2	2.1	4
369	0.56	0.04	0.95	0.17			5
370	0.57	0.05	0.86	0.16			5
373	0.41	0.05	0.80	0.18			5
374	0.31	0.03	0.88	0.07			5
375	0.27	0.01	1.12	0.02			5
376	0.26	0.01	1.02	0.02			5
377	0.33	0.02	1.05	0.03			5
378	0.43	0.03	0.89	0.10			5
381	0.89	0.07	0.05	0.51	12.9	2.8	4
386	0.87	0.06	0.04	0.27	1.5	0.8	4

387	0.77	0.05	1.26	0.39			5
388	1.00	0.03					1
389	0.70	0.02	2.78	0.85			5
391	0.97	0.02	0.26	0.39			2
392	0.89	0.02	0.04	0.01			2
393	0.89	0.03	0.44	0.26			5
394	0.97	0.06	0.70	1.23	1.1	0.6	4
396	0.68	0.02	0.02	0.00			2
397	0.65	0.01	0.59	0.30			5
399	0.65	0.02	1.09	0.28			5
402	0.62	0.02	1.19	0.12			5
403	0.69	0.01	1.03	0.12			5
404	0.69	0.04	0.02	0.00	1.2	0.6	4
405	0.73	0.02	2.09	0.56			5
406	0.85	0.02	0.02	0.01	0.9	0.3	4
408	0.90	0.03	0.02	0.03	0.8	0.4	4
409	0.91	0.02	0.06	0.11			2
410	0.82	0.09			3.6	1.3	3
412	0.94	0.08			5.0	1.2	3
413	0.83	0.04	0.03	0.02			2
414	0.83	0.04	1.02	0.49			5
416	0.95	0.02	0.09	0.27			2
418	1.00	0.05					1
419	0.90	0.02	0.05	0.08			2
420	0.64	0.02	1.70	0.23			5
421	0.66	0.02	0.81	0.30			5
426	0.61	0.02	1.24	0.14			5
429	0.89	0.04	0.07	0.21	3.5	0.7	4
430	0.92	0.06	0.51	0.50	3.0	0.9	4
432	0.86	0.07	0.07	0.32	2.8	1.2	4
434	0.57	0.07	0.79	0.28			5
437	0.21	0.03	0.78	0.04			5
438	0.11	0.01	0.77	0.01			5
439	0.02	0.00	0.66	0.00			5

Table S3. O^2_{axis} parameters and effective internal correlation times for bound TAZ1.

Methyl	O^2_{axis}	O^2_{axis} error	τ_e (s)	τ_e (s) error
341-γ2	0.042	0.001	4.78E-11	1.01E-13
352-δ1	0.426	0.049	5.29E-11	4.16E-12
352-δ2	0.658	0.011	3.82E-11	7.12E-13
353-δ1	0.725	0.028	1.08E-11	1.48E-12
353-γ2	0.764	0.030	1.64E-11	1.58E-12

357-δ1	0.620	0.043	4.78E-11	2.90E-12
357-δ2[a]	0.504	0.117	8.22E-11	1.39E-11
358-γ2	0.665	0.016	2.78E-11	9.89E-13
359-δ2[a]	0.469	0.024	5.13E-11	2.07E-12
360-δ1[a]	0.607	0.037	4.21E-11	2.65E-12
360-δ2	0.734	0.031	5.66E-11	2.45E-12
361-δ1	0.563	0.027	6.74E-11	2.39E-12
361-δ2[a]	0.498	0.119	5.30E-11	1.00E-11
363-β[a]	0.896	0.030	2.54E-11	1.43E-12
378-β[a]	0.487	0.007	4.86E-11	5.82E-13
381-δ1	0.236	0.016	3.84E-11	1.49E-12
387-ε	0.066	0.002	8.92E-12	1.59E-13
390-γ1	0.664	0.015	3.92E-11	9.90E-13
390-γ2	0.620	0.016	3.01E-11	8.93E-13
391-δ1[a]	0.479	0.029	7.82E-11	3.20E-12
391-δ2 [a]	0.456	0.016	4.18E-11	1.15E-12
394-ε	0.331	0.003	1.88E-11	2.17E-13
395-γ2	0.406	0.009	5.48E-11	8.65E-13
399-β [a]	1.00	0.019	4.39E-11	1.72E-12
402-β[a]	0.557	0.012	3.52E-11	7.68E-13
405-γ1	0.831	0.035	8.05E-11	3.24E-12
405-γ2	0.814	0.020	1.82E-11	9.84E-13
406-β	0.843	0.024	5.31E-11	1.68E-12
409-β	0.940	0.040	8.49E-11	3.52E-12
414-γ2	0.759	0.025	2.46E-11	1.35E-12
415-γ2	0.915	0.062	3.65E-11	3.47E-12
422-γ2	0.655	0.040	5.13E-11	3.23E-12
428-γ1	0.749	0.018	5.55E-11	1.38E-12
430-δ1[a]	0.168	0.003	3.53E-11	2.60E-13
430-δ2[a]	0.215	0.003	3.53E-11	3.05E-13
432-δ2[a]	0.198	0.005	4.34E-11	4.87E-13

[a]Obtained from data at 700 MHz (^{1}H) only due to weak peak intensity or overlap at 500 MHz.

Table S4. O^2_{axis} parameters and effective internal correlation times for bound CTAD-HIF-1α. The valine and leucine methyls are not stereospecifically assigned.

Methyl	O^2_{axis}	O^2_{axis} error	τ_e (s)	τ_e (s) error
778-δ1	0.098	0.008	3.97E-11	6.80E-13
779-β	0.236	0.013	5.83E-11	7.58E-13
787-ε	0.070	0.003	1.04E-11	2.43E-13
792-δ2[a]	0.409	0.078	4.48E-11	4.94E-12
796-γ2[a]	0.835	0.125	3.59E-11	5.36E-12
802-γ1	0.538	0.026	3.57E-11	1.68E-12
802-γ2	0.630	0.032	6.12E-11	2.35E-12
804-β[a]	0.610	0.129	4.26E-11	9.21E-12

806-δ1[a]	0.562	0.099	2.63E-11	5.61E-12
806-γ2[a]	0.782	0.104	4.23E-11	5.07E-12
812-δ2	0.363	0.036	4.66E-11	3.47E-12
819-δ1[a]	0.569	0.046	4.27E-11	2.67E-12
819-δ2	0.498	0.040	3.60E-11	2.27E-12
822-δ2[a]	0.978	0.133	3.04E-11	8.75E-12
825-γ1	0.202	0.007	5.43E-11	7.80E-13
825-γ2	0.171	0.007	4.74E-11	7.38E-13

[a]Obtained from data at 700 MHz (^{1}H) only due to weak peak intensity or overlap at 500 MHz.

Table S5. Values of $R(I_zC_zD_z)$, $R(I_zC_zD_y)$, and $R(I_zC_z)$ at 700 and 500 MHz (^{1}H) for bound TAZ1.

**Methyl**	$\boldsymbol{R}$**(I$_z$C$_z$D$_z$) (s^{-1})**	**Error (s^{-1})**	$\boldsymbol{R}$**(I$_z$C$_z$D$_y$) (s^{-1})**	**Error (s^{-1})**	$\boldsymbol{R}$**(I$_z$C$_z$) (s^{-1})**	**Error (s^{-1})**
At 700 MHz (^{1}H):						
341−γ2	21.73	0.04	27.83	0.16	2.22	0.01
352−δ1	25.53	1.96	103.24	10.31	3.80	0.21
352−δ2	21.48	0.27	119.72	1.77	4.73	0.06
353−δ1	13.32	0.56	120.91	4.63	6.60	0.17
353−γ2	14.95	0.59	126.62	4.69	6.10	0.15
357−δ1	25.71	1.11	123.98	6.62	5.89	0.16
357−δ2	40.48	5.50	115.32	16.58	7.13	0.35
358−γ2	17.73	0.39	115.63	2.77	4.48	0.09
359−δ2	26.34	0.79	95.82	3.59	4.70	0.12
360−δ1	24.78	0.96	114.65	5.39	6.42	0.16
360−δ2	30.75	0.95	140.23	4.71	6.36	0.13
361−δ1	33.50	0.90	116.75	4.08	5.78	0.11
361−δ2	28.22	4.00	102.03	17.99	5.89	0.38
363−β	18.41	0.50	151.08	4.35	5.64	0.11
378−β	23.79	0.22	95.96	1.07	3.15	0.05
381−δ1	20.34	0.69	53.66	2.98	3.83	0.18
387−ε	6.78	0.06	16.24	0.30	2.96	0.03
390−γ1	22.49	0.38	122.64	2.35	5.03	0.09
390−γ2	18.65	0.35	110.66	2.33	4.80	0.09
391−δ1	38.48	1.18	109.60	4.16	6.64	0.11
391−δ2	23.15	0.45	90.68	2.24	5.16	0.10
394−ε	12.30	0.09	61.13	0.52	3.80	0.04
395−γ2	27.39	0.36	87.78	1.34	4.89	0.06
399−β	24.63	0.62	183.21	4.83	5.08	0.11
402−β	18.52	0.29	100.97	1.79	2.90	0.08
405−γ1	38.60	1.25	162.91	5.65	5.45	0.11
405−γ2	13.11	0.38	135.99	3.23	3.52	0.12
406−β	27.18	0.65	155.17	3.92	4.51	0.10

409–β	41.54	1.41	179.51	6.88	6.60	0.13
414–γ2	18.00	0.52	131.14	3.93	5.89	0.13
415–γ2	23.60	1.38	169.60	11.36	7.57	0.21
422–γ2	24.97	1.47	123.53	7.93	3.51	0.16
428–γ1	28.98	0.54	139.09	2.73	5.41	0.08
430–δ1	17.54	0.10	42.48	0.46	2.69	0.03
430–δ2	18.15	0.11	50.06	0.49	3.18	0.03
432–δ2	21.67	0.18	51.06	0.76	3.53	0.05
At 500 MHz (^{1}H):						
341–γ2	24.22	0.13	30.43	0.46	2.66	0.04
352–δ1	30.46	2.95	80.00	9.93	5.74	0.38
352–δ2	26.46	0.72	118.49	4.29	5.43	0.14
353–δ1	17.15	1.45	123.24	10.86	7.26	0.41
353–γ2	19.32	1.57	140.54	11.63	6.24	0.38
357–δ1	36.33	3.39	93.64	14.33	6.61	0.40
358–γ2	19.27	0.91	122.44	6.24	4.94	0.22
360–δ2	30.87	2.22	136.58	12.72	6.67	0.32
361–δ1	40.71	5.60	130.78	24.51	6.25	0.42
381–δ1	20.89	0.94	58.67	3.64	4.36	0.21
387–ε	7.82	0.15	18.96	0.82	3.14	0.09
390–γ1	24.61	1.09	110.26	6.66	5.41	0.26
390–γ2	20.57	0.87	111.87	5.44	4.63	0.23
394–ε	15.95	0.26	64.93	1.56	4.02	0.11
395–γ2	32.00	0.83	90.69	4.00	5.27	0.15
405–γ1	40.16	2.61	160.69	11.51	6.15	0.25
405–γ2	18.12	0.90	127.84	6.57	3.98	0.25
406–β	31.36	1.48	143.62	8.13	4.79	0.23
409–β	42.05	3.20	193.48	17.88	7.43	0.31
414–γ2	20.65	1.26	130.30	9.26	5.92	0.32
415–γ2	31.85	2.94	133.69	19.34	7.77	0.44
422–γ2	28.85	1.98	123.58	10.04	3.83	0.24
428–γ1	32.49	1.28	150.33	7.46	6.09	0.20

Table S6. Values of $R(I_zC_zD_z)$, $R(I_zC_zD_y)$, and $R(I_zC_z)$ at 700 and 500 MHz (^{1}H) for bound CTAD-HIF-1α.

**Methyl**	$\boldsymbol{R}$**(I$_z$C$_z$D$_z$) (s^{-1})**	**Error (s^{-1})**	$\boldsymbol{R}$**(I$_z$C$_z$D$_y$) (s^{-1})**	**Error (s^{-1})**	$\boldsymbol{R}$**(I$_z$C$_z$) (s^{-1})**	**Error (s^{-1})**
At 700 MHz (^{1}H):						
778–δ1	18.86	0.28	31.63	1.37	2.89	0.20
779–β	25.94	0.36	60.20	2.29	3.40	0.24
787–ε	5.26	0.09	14.88	0.47	0.96	0.11
792–δ2	24.11	1.78	84.72	10.98	5.04	0.61

796–γ2	21.14	1.78	144.78	21.39	4.66	0.77
802–γ1	19.07	0.74	103.42	5.00	4.44	0.36
802–γ2	28.77	1.10	119.52	6.46	5.53	0.34
804–β	24.23	3.30	114.59	19.50	5.66	0.86
806–δ1	19.53	1.86	102.71	14.29	7.28	0.82
806–γ2	25.03	1.74	140.79	14.55	6.28	0.64
812–δ2	23.13	1.52	90.26	7.58	6.46	0.61
819–δ1	23.65	0.95	107.99	6.70	5.13	0.39
819–δ2	20.67	1.12	93.93	7.50	5.63	0.49
822–δ2	22.60	3.08	167.42	34.52	7.81	1.13
825–γ1	24.22	0.33	53.91	1.20	3.10	0.15
825–γ2	21.98	0.34	47.20	1.29	3.36	0.18
At 500 MHz (^{1}H):						
778–δ1	20.41	0.34	38.89	2.01	2.86	0.20
779–β	29.70	0.36	66.90	3.61	3.42	0.17
787–ε	5.86	0.08	17.70	0.63	0.91	0.11
802–γ1	23.11	0.84	96.75	5.41	3.84	0.44
802–γ2	35.57	1.24	130.21	6.95	4.64	0.39
812–δ2	32.75	2.12	72.23	6.70	5.97	0.79
819–δ2	23.78	1.18	98.05	8.68	5.29	0.57
825–γ1	29.60	0.47	59.14	1.57	3.78	0.19
825–γ2	25.69	0.44	50.44	1.50	3.43	0.25

References

1. Lindström, I. & Dogan, J. (2018) Dynamics, Conformational Entropy, and Frustration in Protein-Protein Interactions Involving an Intrinsically Disordered Protein Domain, *ACS Chem Biol.* **13**, 1218-1227.
